# Efficacy of Humanized Anti-BCMA CAR T Cell Therapy in Relapsed/Refractory Multiple Myeloma Patients With and Without Extramedullary Disease

**DOI:** 10.3389/fimmu.2021.720571

**Published:** 2021-08-05

**Authors:** Haobin Deng, Meijing Liu, Ting Yuan, Huan Zhang, Rui Cui, Jingyi Li, Jijun Yuan, Xiaofang Wang, Yafei Wang, Qi Deng

**Affiliations:** ^1^First Central Clinical College, Tianjin Medical University, Tianjin, China; ^2^Department of Hematology, Tianjin First Central Hospital, School of Medicine, Nankai University, Tianjin, China; ^3^Shanghai Genbase Biotechnology Co., Ltd., Shanghai, China; ^4^Department of Hematology, Tianjin Medical University Cancer Institute and Hospital, Tianjin, China

**Keywords:** anti-B cell maturation antigen chimeric antigen receptor T, multiple myeloma, relapsed, refractory, extramedullary disease, efficacy

## Abstract

In recent years, many new treatments for relapsed/refractory (R/R) multiple myeloma (MM) have improved patient prognosis, but the prognosis of patients with extramedullary MM is still particularly poor. Therefore, more efficacious therapies and novel strategies are urgently needed for these patients. The aim of this study was to observe and compare the efficacy and safety of humanized anti-B cell maturation antigen (anti-BCMA) chimeric antigen receptor (CAR) T cell therapy in R/R MM patients with and without extramedullary disease. Seven R/R MM patients with extramedullary disease and 13 without extramedullary disease received humanized anti-BCMA CAR T cell therapy. The overall response rate was not different between patients with and without extramedullary disease. There was no difference in the progression-free survival (PFS) or overall survival (OS) rates between the two groups at 180 days, but the PFS and OS rates in patients with extramedullary disease were lower at 360 days than those in patients without extramedullary disease. Although some patients with extramedullary disease experienced further disease progression, their M protein level did not increase. We did not see this change trend of M protein in patients without extramedullary disease. However, this was not observed in patients without extramedullary disease. Among patients who responded to CAR T cell therapy, the grades of cytokine release syndrome (CRS) and immune effector cell-associated neurotoxic syndrome (ICANS) were much higher among patients with extramedullary disease. In summary, R/R MM patients with extramedullary disease could benefit from humanized anti-BCMA CAR T cell therapy in the short term, although the CRS and ICANS grades were much higher in patients with extramedullary disease. Therefore, anti-BCMA CAR T cell therapy allows for a remission time for R/R MM patients with extramedullary disease, which could be maintained by bridging hematopoietic stem cell transplantation, radiotherapy, and other therapies.

**Clinical Trial Registration:**

http://www.chictr.org.cn/index.aspx, identifiers ChiCTR1800017051 and ChiCTR2000033925.

## Introduction

Multiple myeloma (MM) is a malignant disease characterized by monoclonal proliferation of bone marrow (BM) plasma cells. Although effective treatment, such as proteasome inhibitors, immunomodulatory agents, monoclonal antibodies, and autologous hematopoietic stem cell transplantation (auto-HSCT), can significantly improve patient prognosis (1–4), the prognosis of patients with relapsed/refractory (R/R) MM is poor (5, 6). Extramedullary MM (EMM) is a rare type of MM in which MM cells escape the BM, become independent of the BM microenvironment, and invade other organs, resulting in extramedullary disease. Any organ can be infiltrated, including the skin, soft tissues, liver, spleen, kidney, lymph nodes, breast, pleura, and central nervous system (7, 8). The molecular mechanisms underlying EMM development have not yet been defined. The prognosis of EMM is different from that of MM without extramedullary disease (9). EMM is associated with an increased risk and poor prognosis, and many treatments, including ASCT, have failed to improve patient outcomes in most studies (10). In recent years, many new treatments for R/R MM have improved patient prognosis, but the prognosis of patients with EMM is still particularly poor (11–15). Therefore, more efficacious therapies and novel strategies are urgently needed for EMM.

Anti-B cell maturation antigen (anti-BCMA) is specifically expressed in the cells of almost all MM patients (16). Anti-BCMA chimeric antigen receptor (CAR) T cell therapy had satisfactory efficacy and prolonged the survival time of R/R MM patients in early clinical trials (17–20). However, there are limited data on anti-BCMA CAR T cell therapy in patients with EMM. Whether anti-BCMA CAR T cell therapy could improve the outcomes of R/R MM patients with extramedullary disease needs to be further explored.

## Patients and Methods

### Patients

Twenty R/R MM patients were enrolled in a clinical trial of humanized anti-BCMA CAR T cell therapy (*ChiCTR1800017051* and *ChiCTR2000033925*) between January 2019 and October 2020. These 20 R/R MM patients included seven patients with at least one assessable extramedullary lesion and 13 patients without any assessable extramedullary disease. The extramedullary disease in the seven patients was classified as either extramedullary-extraosseous (EM-E) or extramedullary-bone related (EM-B). None of the seven patients had solitary extramedullary/bone plasmacytoma.

### Preparation and Administration of Humanized Anti-BCMA CAR T Cell Therapy

Peripheral blood mononuclear cells were collected from R/R MM patients and isolated by Ficoll density gradient centrifugation. CD3+ T cells were selected using CD3 microbeads (Miltenyi Biotec, Inc., Cambridge, MA, USA), stimulated by anti-CD3/anti-CD28 mAb-coated Human T-Expander beads (Cat. no. 11141D, Thermo Fisher Scientific, Inc., Waltham, MA, USA), and cultured in X-Vivo 15 medium (Lonza Group, Ltd., Basel, Switzerland) supplemented with 250 IU/ml interleukin (IL)-2 (Proleukin, Novartis International AG, Basel, Switzerland). CD3+ T cells (3 × 10^6^) were transduced with lentiviral supernatant from 293T cells transfected with anti-BCMA CAR plasmid (20 µg, lenti-BCMA-2rd-CAR, Shanghai Genbase Biotechnology Co., Ltd. Shanghai, China) at a multiplicity of infection of 0.5 and cultured in media containing IL-2 (250 U/ml). On the 12th to 15th days of cultivation, transduction efficiencies of anti-BCMA CAR were analyzed by flow cytometry (FCM) (BD Biosciences, San Jose, CA, USA).

All patients received lymphodepleting chemotherapy with fludarabine (30 mg/m^2^) and cyclophosphamide (400 mg/m^2^) from day −4 to day −2. Autologous anti-BCMA CAR T cells were infused on day 0 (2 × 10^6^ cells/kg) in all patients.

### Evaluation Criteria for Diagnostic and Therapeutic Efficacy

The diagnosis of EMM, clinical response, and disease progression after humanized anti-BCMA CAR T cell therapy were assessed according to the International Myeloma Working Group Guidelines uniform response criteria for MM (21). Follow-up was performed from the date of humanized anti-BCMA CAR T cell infusion until the cutoff date or until the patient died. The clinical responses included stringent complete response (sCR), complete response (CR), very good partial response (VGPR), partial response (PR), minimal response, stable disease (SD), and progressive disease. In our study, we assessed the objective response rate (ORR), overall survival (OS), and progression-free survival (PFS). The ORR was defined as the proportion of patients who achieved sCR, CR, VGPR, or PR.

Assessable extramedullary disease was detected using computed tomography. MM cells in the pleural effusion and cerebrospinal fluid were detected using FCM. During humanized anti-BCMA CAR T cell therapy administration, the proportions of anti-BCMA CAR T cells in the peripheral blood were assessed by FCM on days 0, 4, 7, 14, 28, and 60 after infusion of anti-BCMA CAR T cells. Proportions of anti-BCMA CAR T cells in the pleural effusion and cerebrospinal fluid were also observed by FCM.

### Adverse Events (AEs) of Humanized Anti-BCMA CAR T Cell Therapy

AEs of humanized anti-BCMA CAR T cell therapy were also assessed. The cytokine release syndrome (CRS) grade was determined according to the National Cancer Institute Common Terminology Criteria for Adverse Events v4.03 (22). Immune effector cell-associated neurotoxicity syndrome (ICANS) was used to assess neurotoxicity (23). Cytokines, including IL-6, IL-2R, and tumor necrosis factor-α (TNF-α) were assessed on days 0, 7, 14, 28, and 60 by enzyme-linked immunosorbent assay.

### Statistical Analysis

Data are expressed as the mean ± SE. The probabilities of PFS and OS were estimated using the Kaplan-Meier method and compared with the log-rank test. All statistical analyses were performed using GraphPad Prism 8 and SPSS 17.0. Statistical significance was set at *P* < 0.05.

## Results

### Patient Characteristics

The characteristics of all R/R MM patients enrolled in this clinical trial are listed in **Table 1**. BCMA was expressed on MM cells in all the R/R MM patients when they were enrolled in our study (**Table 1**). The expressions of BCMA in MM cells by FCM at the time of enrollment are shown in **Supplementary Figure 1**. The only difference between the two groups was the presence of extramedullary disease (**Table 2**). In our study, two patients in the EMM group had prior anti-BCMA CAR-T cell therapy. These two patients received murine anti-BCMA CAR-T cell therapy before, but they did not benefit from this time treatment. So they were enrolled in this humanized anti-BCMA CAR-T cell therapy. In the seven R/R MM patients with extramedullary disease, the mean time from diagnosis of extramedullary disease to enrolment in this clinical trial was 9.57 ± 3.08 months. All the patients had BCMA expression in MM cells at the time of enrolment. The median follow-up times were 7.3 months (range, 2–12 months) in patients with extramedullary disease and 12.5 months (3–27 months) in patients without extramedullary disease.

**Table 1:** Baseline characteristics of the R/R multiple myeloma patients with or without extramedullary disease.

Pt	Sex	Age (years)	KPS	Subtype	ISS stage	MM cells in the BM, %	Bone lesions	Extramedullary disease	Time to diagnosis of extramedullary disease (months)	High-risk cytogenetic lesions	Lines of prior therapy	Previous BCMA CAR T cell therapy (Murine BCMA CAR T)	Previous auto-HSCT	BCMA expression in MM cells (%)
***With extramedullary disease***														
1	Male	57	90	κ light chain	III	86.0%	Yes	EM-B/EM-E	5	t(4;14), t(14;16), Del(17p)	14	No	Yes	92.53
2	Female	71	100	IgG-κ	II	55.3%	Yes	EM-B/EM-E	14	Del(17p),t(4;14)	10	Yes	No	94.39
3	Male	59	100	κ light chain	III	17.0%	Yes	EM-B/EM-E	9	t(14,16)	7	Yes	No	81.82
4	Male	73	90	IgD-λ	II	23.04%	Yes	EM-E	3	None	12	No	No	94.39
5	Female	38	90	κ light chain	II	70.44%	Yes	EM-B/EM-E	6	None	17	No	Yes	93.18
6	Male	56	80	IgG-κ	II	32.26%	Yes	EM-B/EM-E	5	None	10	No	No	80.26
7	Female	58	90	IgG-λ	III	88.12%	Yes	EM-E	12	None	30	No	No	93.68
***Without extramedullary disease***														
1	Female	70	100	IgG-κ	II	32.56%	Yes	-	-	Del(17p), t(4;14)	10	No	No	94.76
2	Male	58	100	κ light chain	III	41.75%	Yes	-	-	t(14,16)	7	No	No	92.57
3	Male	63	80	IgG-κ	III	35.62%	Yes	-	-	t(14,16)	5	No	Yes	97.84
4	Male	66	100	IgA-κ	II	19.2%	Yes	-	-	t(14;16), Del(17p)	16	No	No	90.99
5	Female	55	90	IgG-λ	II	44.28%	No	-	-	None	7	No	No	90.41
6	Female	52	90	IgA-λ	II	20.72%	Yes	-	-	None	9	No	No	82.93
7	Female	72	100	Nonsecretory	II	28.98%	Yes	-	-	None	6	No	No	95.70
8	Female	77	90	IgD-κ	III	66.8%	Yes	-	-	None	13	No	No	97.06
9	Female	42	100	IgG-κ	III	22.14%	Yes	-	-	t(14;16)	5	No	No	96.24
10	Female	45	90	IgG-κ	I	19.44%	Yes	-	-	None	5	No	No	82.73
11	Female	46	90	IgG-κ	III	22.23%	Yes	-	-	None	7	No	No	88.83
12	Male	54	90	IgG-κ	III	17.5%	Yes	-	-	None	8	No	No	92.31
13	Male	54	80	IgG-λ	II	14.8%	Yes	-	-	t(14;16)	9	No	Yes	88.16

Pt, Patient; KPS, Karnofsky performance scale; ISS, international staging system; MM, multiple myeloma; BM, bone marrow; BMCA, B cell maturation antigen; CAR, chimeric antigen receptor; auto-HSCT, autologous haematopoietic stem cell transplantation; EM-B, extramedullary-bone related; EM-E, extramedullary-extraosseou.

Extramedullary-extraosseous (EM-E): Hematogenous dissemination leads to soft tissue tumors at the anatomical site far from the bone.

Extramedullary-bone related (EM-B): Breaks through the bone cortex and only invade the surrounding soft tissues.

**Table 2:** To compare the baseline characteristics of the R/R multiple myeloma patients with or without extramedullary disease.

	With extramedullary disease (n = 7)	Without extramedullary disease (n = 13)	*P*
**Sex, male**	4 (57.1%)	8 (61.5%)	>0.99
**Age, years**	58 (38-73)	58 (42-77)	0.964
**KPS >80**	7 (100%)	13 (100%)	0.796
**Subtype, %κ light chain**	42.9%	7.7%	0.101
**ISS stage, I-II:III (n)**	4:7	7:6	0.444
**MM cells in the BM, %, mean (range)**	36.6 (7.8-86.0)	14.8 (1.8-66.7)	0.059
**Bone lesions**	7 (100%)	11 (84.6%)	0.521
**Extramedullary MM**	7 (100%)	0 (0%)	**<0.0001**
**Extramedullary-extraosseous**	6 (85.7%)	0 (0%)	**0.0002**
**Extramedullary-bone related**	5 (71.5%)	0 (0%)	**0.001**
**High-risk cytogenetic lesions**	3 (42.9%)	6 (46.2%)	>0.99
**Lines of prior therapy, mean (range)**	12 (7-30)	7 (5-16)	0.085
	2 (28.6%)	0 (0%)	0.111
**Auto-HSCT before BCMA CAR T therapy**	2 (28.6%)	2 (15.4%)	0.587

Data are presented as n (%) unless otherwise indicated.

ISS, international staging system; MM, multiple myeloma; BM, bone marrow; auto-HSCT, autologous haematopoietic stem cell therapy; CAR, chimeric antigen receptor.

In bold: The only difference between the two groups was the presence of extramedullary disease (P < 0.05).

### Transduction Efficiency, Amplification Efficiency, and Infusion Dose of Humanized Anti-BCMA CAR T Cells

The median humanized anti-BCMA CAR transduction efficiencies were 38.74 ± 9.18% in the extramedullary disease group and 42.26 ± 10.41% in the no extramedullary disease group (*P* = 0.826). After 12 to 15 days of culture, the median humanized anti-BCMA CAR T cell numbers were 5.87 ± 2.77 × 10^6^ cells/kg and 5.01 ± 1.94 × 10^6^ cells/kg in the extramedullary disease and no extramedullary disease groups, respectively (*P* = 0.799). The patients received anti-BCMA CAR T cell doses of 2.21 ± 0.39 × 10^6^ cells/kg and 2.06 ± 0.44 × 10^6^ cells/kg, respectively, on day 0 (*P* = 0.825). Manufactured products and phenotypical analysis of the final formulation per patient are in **Supplementary Table 1**.

### Clinical Response and Survival

The median response times to achieve the best effect were 2.7 months (1–6 months) in the extramedullary disease group and 2.3 months (1–4 months) in the no extramedullary disease group. There was no difference in the median response time between the two groups (*P* = 0.967).

**Figure 1:**
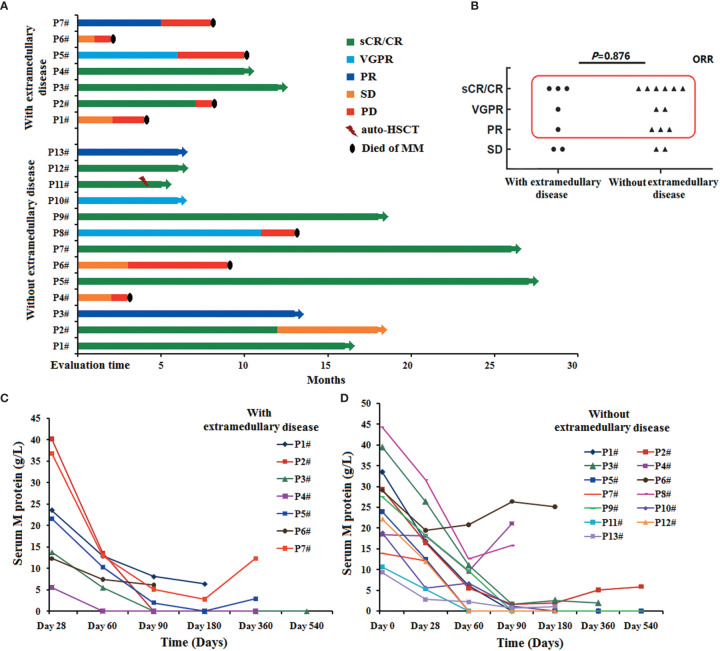
Clinical responses of the humanized anti-BCMA CAR-T cell therapy. **(A)** Pt_with_ 1,2,5,6,7 (5/7, 71.4%) and Pt_without_ 4,6,8 (3/13, 23.1%) developed disease progression again and died of their primary disease. **(B)** There was no difference in ORR between patients with and without extramedullary disease when they achieved the best effect (*P*=0.876). **(C)** M protein levels increased again in Pt_with_ 5,7 when their disease progressed again, but it did not increase when the disease of Pt_with_ 1,2,6 progressed again. **(D)** All the patients with disease progression were accompanied by an increase of M protein level.

Five patients in the extramedullary disease group (Pt_with_ 1, 2, 5, 6, and 7) (71.4%) experienced further disease progression and died of their primary disease. Only three patients in the no extramedullary disease group (Pt_without_ 4, 6, and 8) (3/13, 23.1%) experienced disease progression and died of their primary disease. Pt_without_ 2 also experienced further disease progression, but he was still alive at the cutoff date (**Figure 1A**). When the disease progress again in all the patients, the anti-BCMA expressions in myeloma cells was positive in Pt_with_ 2,5,7 and Pt_without_ 2,4, whereas it was negative in Pt_with_ 1,6 and Pt_without_ 6,8.

After this anti-BCMA CAR-T cell therapy, the ORR in all the R/R MM patients was 80% (16/20). In all the seven patients with extramedullary disease, five patients had an ORR reaction to this therapy (71.43%, 5/7), Of the seven patients with extramedullary disease, five responded to CAR T cell therapy, including three patients who achieved sCR/CR, one patient who achieved VGPR, and one patient who achieved PR. The other two patients achieved SD only. In the patients without extramedullary disease, 11 patients had an ORR reaction to this therapy (84.61%, 11/13), Of the patients without extramedullary disease, 11 patients responded to CAR T cell therapy, including six patients who achieved sCR/CR, two patients who achieved VGPR, and three patients who achieved PR. The other two patients achieved SD only (**Figure 1B**). There was no difference in the ORR between patients with and without extramedullary disease when they achieved the best effect (*P* = 0.876). Only one patient without extramedullary disease (Pt_without_ 11) received auto-HSCT 3 months after anti-BCMA CAR T-cell therapy.

The M protein levels in the peripheral blood were assessed following anti-BCMA CAR T-cell infusion. In the seven patients with extramedullary disease, the level of M protein increased again in Pt_with_ 5 and 7 after disease progression (**Figures 1C, D** **)**. However, there was no increase in the M protein levels of Pt_with_ 1 and 2 after disease progression. All patients without extramedullary disease who experienced disease progression had an increase in M protein levels (**Figure 1D**). We also assessed the anti-BCMA expression in MM cells among patients with disease progression. We observed anti-BCMA expression in the MM cells of Pt_with_ 2, 5, and 7 and Pt_without_ 2 and 4 but not those of Pt_with_ 1 and 6 and Pt_without_ 6 and 8.

**Figure 2:**
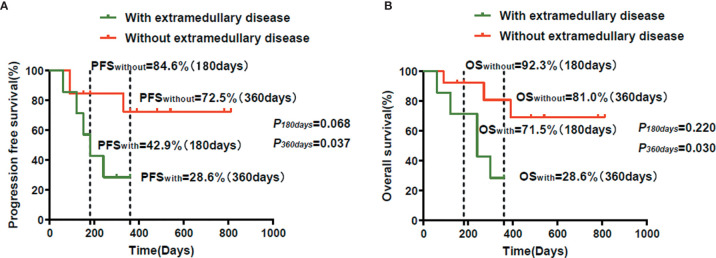
The survival observation of the humanized anti-BCMA CAR-T cell therapy. **(A)** The PFS in patients with extramedullary disease was lower than that of the patients without extramedullary disease at 360 days (*P*=0.037). There was no difference of the PFS in the two groups at 180 days (*P*=0.068). **(B)** The OS in patients with extramedullary disease was lower than that of the patients without extramedullary disease at 360 days (*P*=0.030). There was no difference of the OS in the two groups at 180 days (*P*=0.220).

Kaplan-Meier analysis showed that there was no difference in the PFS rate between patients with extramedullary disease (42.9%) and those without extramedullary disease (84.6%) at 180 days (*P* = 0.068). However, the PFS rate of patients with extramedullary disease (28.6%) was lower than that of patients without extramedullary disease (72.5%) at 360 days (*P* = 0.037) (**Figure 2A**). There was no difference in the OS rate between patients with extramedullary disease (71.5%) and those without extramedullary disease (92.3%) at 180 days (*P* = 0.220). However, the OS rate of patients with extramedullary disease (28.6%) was lower than that of patients without extramedullary disease (81.0%) at 360 days (*P* = 0.030) (**Figure 2B**).

In our study, the mean OS among patients with extramedullary disease was 17.29 ± 4.95 months.

### Efficacy of Humanized Anti-BCMA CAR T Cell Therapy in Patients With EMM

We evaluated the efficacy of humanized anti-BCMA CAR T-cell therapy in the seven patients with extramedullary disease, including the efficacy for extramedullary lesions. At the beginning of the study, Pt_with_ 4 had skin lesions with no extramedullary bone-related disease in the adjacent bone, Pt_with_ 7 had myeloma cells infiltrating the pleural effusion and central nervous system MM, and Pt_with_ 5 had a number of extramedullary bone-related lesions. The four remaining patients all had at least one extramedullary soft tissue lesion far from the bone.

**Figure 3:**
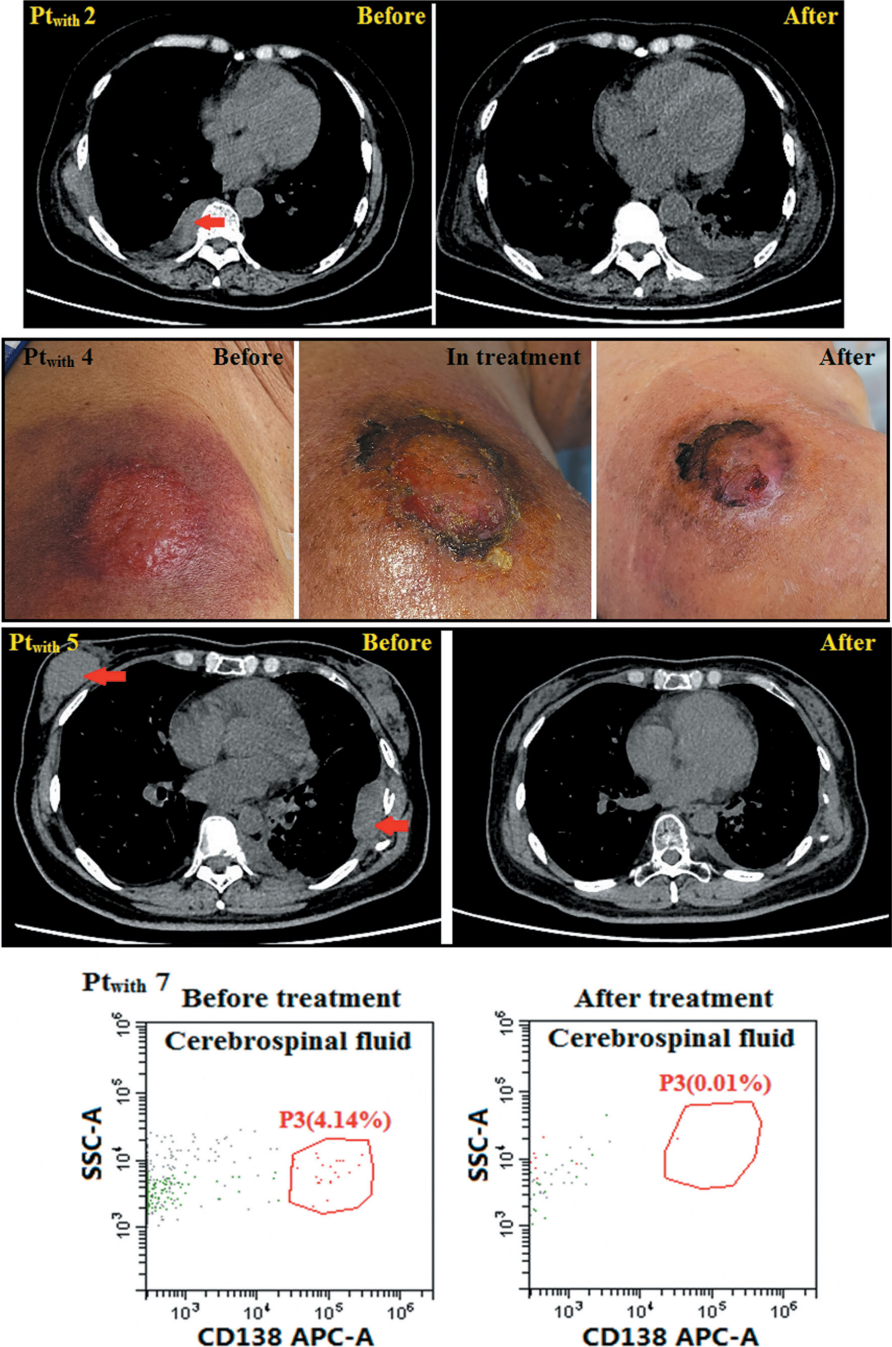
The efficacy to the extramedullary disease. The skin lesion far from bone of Pt_with_ 4 disappeared after anti-BCMA CAR-T cell therapy. Myeloma cells in cerebrospinal fluid of Pt_with_ 7 disappeared after therapy. Soft tissue masses disappeared after this therapy.

Although the M protein in the peripheral blood of Pt_with_ 6 disappeared after treatment, as did myeloma cells in the BM, he did not achieve sCR/CR as of the cutoff date because he had extramedullary lesions that did not disappear after humanized anti-BCMA CAR T cell therapy. The skin lesion far from the bone of Pt_with_ 4 disappeared after anti-BCMA CAR T cell therapy. The myeloma cells in the cerebrospinal fluid of Pt_with_ 7 disappeared after therapy. The soft tissue masses in the remaining four patients with extramedullary disease disappeared after anti-BCMA CAR T cell therapy (**Figure 3**).

### The Proportions of Humanized Anti-BCMA CAR T Cells

**Figure 4:**
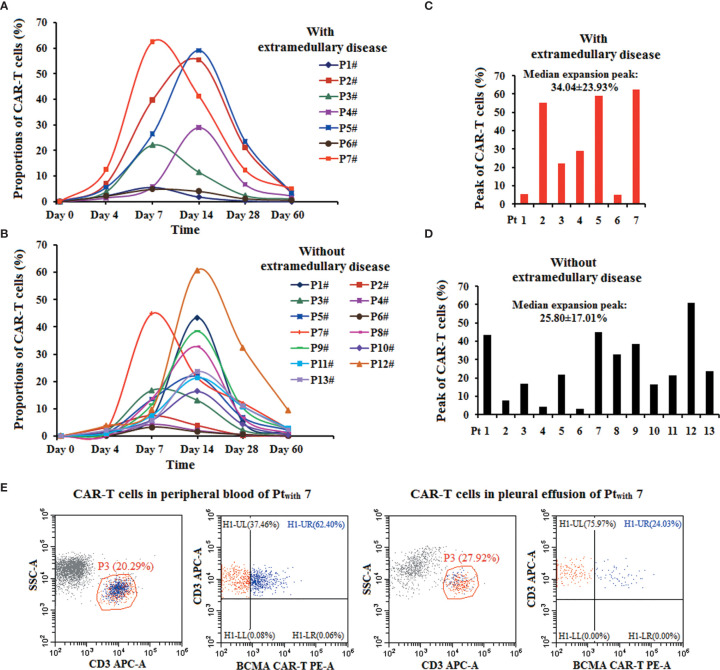
The proportions of the anti-BCMA CAR-T cells in the therapy. **(A, B)**. The proportions of anti-BCMA-CAR T cells in CD3+ T cells in peripheral blood in the two group of patients. **(C, D)**. There was no difference in the median expansion peak of the anti-BCMA CAR-T cells in CD3+ T cells in peripheral blood in the two group of patients (*P*= 0.438). **(E)**. In Pt_with_ 7, the highest anti-BCMA CAR-T cell expression in peripheral blood was 62.4%, the level of anti-BCMA CAR-T cells in her pleural effusion was 24.03%.

The proportion of CAR T cells was assessed on days 0, 4, 7, 14, 28, and 60 post-infusion. There was no difference in the median expansion peak of the humanized anti-BCMA CAR T cells in the peripheral blood of patients with extramedullary disease (34.04 ± 23.93%) and those without extramedullary disease (25.80 ± 17.01%) (*P* = 0.438) (**Figures 4A–D**). The highest anti-BCMA CAR T cell expression in the peripheral blood occurred in Pt_with_ 7 (62.4%), and the level of anti-BCMA CAR T cells in her pleural effusion was 24.03% (**Figure 4E**).

### AEs and Serum Levels

**Figure 5:**
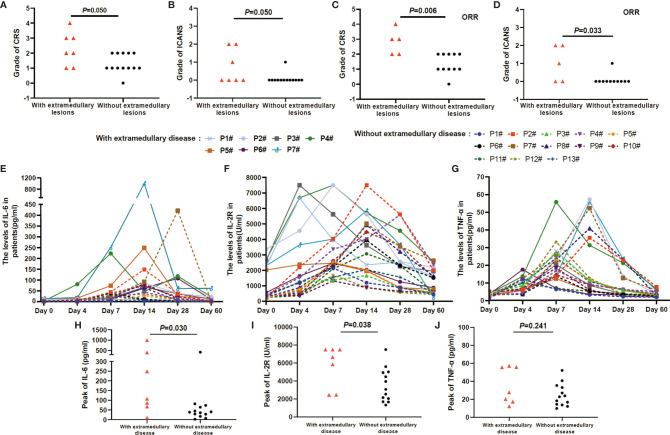
AEs and the serum levels in the anti-BCMA-CAR T cell therapy. **(A, B)** The grades of CRS and ICANS had no different in patients with and without extramedullary disease (*P* ^CRS^ = 0.050 and *P* ^ICANS^ = 0.050). **(C, D)** In patients obtained an ORR reaction, the grades of CRS and ICANS in patients with extramedullary disease was higher than that of in patients without extramedullary disease (*P* ^CRS^ =0.006 and *P* ^ICANS^ = 0.033). **(E–G)** The serum levels of IL-6, IL-2R, and TNF-a peaked 4 to 7 days after the infusion of anti-BCMA CAR T cells and declined 12 to 21 days. **(H, I)** IL-6 and IL-2R levels were higher in patients with extramedullary disease group than that of in patients without extramedullary disease group (*P* ^IL-6^ = 0.030 and *P* ^IL-2R^ = 0.038). **(J)** There was no different in the TNF-a level between the two groups (*P* ^TNF-a^ = 0.241).

Pt_with_ 7 was diagnosed with grade 4 CRS, Pt_with_ 2 and 5 were diagnosed with grade 3 CRS, and the remaining patients in both groups were diagnosed with grades 0 to 2 CRS. Pt_with_ 2 and 7 were diagnosed with grade 2 ICANS, Pt_with_ 2 and Pt_without_ 8 were diagnosed with grade 1 ICANS, and the remaining patients in both groups were diagnosed with grade 0 ICANS. The proportions of the grades of CRS and ICANS did not differ between the two groups (*P*_CRS_ = 0.050 and *P*_ICANS_ = 0.050) (**Figures 5A, B**). However, when including only patients who responded to CAR T cell therapy, the grades of CRS and ICANS in patients with extramedullary disease were significantly higher than those in patients without extramedullary disease (*P*_CRS_ = 0.006 and *P*_ICANS_ = 0.033) (**Figures 5C, D**). No patient died of any grade CRS or ICANS during therapy. CRS grade >3 was more frequent in patients with extramedullary disease than in those without extramedullary disease (*P* = 0.031) (**Table 3**).

**Table 3:** Adverse events in the anti-BCMA-CAR T cell therapy.

	With extramedullary disease (n=7)	Without extramedullary disease (n=13)	*P values*
**CRS**			
**Grade 0-2**	4 (57.1%)	13 (100.0%)	
**Grade ≥3**	3 (42.9%)	0 (0%)	***P*=0.031**
**ICANS**			
**Grade 0-2**	7 (100.0%)	13 (100.0%)	
**Grade≥3**	0	0	**-**
**Coagulopathy**			
**Grade 0-2**	5 (71.4%)	12 (92.3%)	
**Grade≥3**	2 (28.6%)	1 (7.6%)	*P*=0.270
**Gastrointestinal**			
**Grade 0-2**	6 (85.7%)	13 (100.0%)	
**Grade≥3**	1 (14.3%)	0	*P*=0.350
**Creatinine increased**			
**Grade 0-2**	7 (100.0%)	13 (100.0%)	
**Grade≥3**	0	0	**-**
**Transaminase increases**			
**Grade 0-2**	5 (71.4%)	13 (100.0%)	
**Grade≥3**	2 (28.6%)	0	*P*=0.111
**Cardiopulmonary**			
**Grade 0-2**	6 (85.7%)	13 (100.0%)	
**Grade≥3**	1 (14.3%)	0	*P*=0.350
**Hematological toxicity**			
***Leukopenia***			
**Grade 0-2**	4 (57.1%)	11 (84.6%)	
**Grade≥3**	3 (42.9%)	2 (15.4%)	*P*=0.290
***Anemia***			
**Grade 0-2**	5 (71.4%)	10 (76.9%)	
**Grade≥3**	2 (28.6%)	3 (23.1%)	*P*=1.000
***Thrombocytopenia***			
**Grade 0-2**	3 (42.9%)	11 (84.6%)	
**Grade≥3**	4 (57.1%)	2 (15.4%)	*P*=0.122

Data are presented as n (%).

CRS, cytokine release syndrome; ICANS, immune effector cell-associated neurotoxic syndrome.

In bold: CRS grade >3 was more frequent in patients with extramedullary disease than in those without extramedullary disease (P = 0.031).

The cytokine levels of IL-6, IL-2R, and TNF-α changes during humanized anti-BCMA CAR T-cell therapy (**Figures 5E–G**). In all of the patients, the serum levels of IL-6, IL-2R, and TNF-α peaked 4 to 7 days after the infusion of anti-BCMA CAR T cells and declined 12 to 21 days after infusion. The serum levels of IL-6 and IL-2R were higher in patients with extramedullary disease than in those without extramedullary disease (*P*_IL-6_ = 0.030 and *P*_IL-2R_ = 0.038) (**Figures 5H, I**). However, there was no difference in the serum level of TNF-α between patients with and without extramedullary disease (*P*_TNF-α_ = 0.241) (**Figure 5J**).

Patients developed fever with or without chills, headache, fatigue, nausea, reduced appetite, edema, tachycardia, and other symptoms during humanized anti-BCMA CAR T cell therapy (**Table 3**). All of the patients recovered 14 to 28 days after the infusion of anti-BCMA CAR T cells. Patients also experienced grades 1 to 4 hematological toxicities in the course of anti-BCMA CAR T-cell therapy. These toxicities occurred 5 to 8 days after infusion of anti-BCMA CAR T cells, and patients recovered 14 to 60 days after infusion. The patients received tocilizumab, methylprednisolone, antipyretic drugs, and symptomatic treatment for AEs. Three patients with extramedullary disease and two patients without extramedullary disease who presented with grade 3 hematological toxicity were diagnosed with gram-negative bacterial infections, which were cured by anti-infective therapy. No patient was diagnosed with invasive fungal disease, and none of the patients died of infections.

## Discussion

Seven R/R MM patients with assessable extramedullary disease and 13 R/R MM patients without extramedullary disease were enrolled in this clinical trial of humanized anti-BCMA CAR T-cell therapy. The process of observation after anti-BCMA CAR T cell therapy was comprehensive rather than just observing the changes in M protein levels. Although the grades of CRS and ICANS were much higher in patients with extramedullary disease, the ORR was not different in patients with and without extramedullary disease, and there was no difference between the two groups in the PFS and OS rates at 180 days. These results suggest that R/R MM patients with extramedullary disease who respond to anti-BCMA CAR T cell therapy can use this time as a bridge to other treatments, such as HSCT, radiotherapy, and other therapies.

MM is a malignant B-cell disease associated with organ damage, including renal failure, anemia, bone damage, and hypercalcemia (21, 24). EMM is defined as the proliferation of malignant plasma cells outside the BM. The EMM incidence is approximately 10% to 15% in all R/R MM patients (25, 26). Primary EMM occurs in approximately 4% to 16% of MM patients at diagnosis, and secondary EMM occurs in approximately 6% to 20% of MM patients during disease progression (27). There are two types of EMM. EM-E occurs when hematogenous dissemination leads to soft tissue tumors at a site far from the bone, and EM-B involves MM cells breaking through the bone cortex and invading only the surrounding soft tissues (9, 28–31). EM-E occurs in approximately 3% of MM patients who experience relapse, whereas EM-B occurs in 6%-34% of patients (30, 32). In our study, 7 of 20 R/R MM patients had at least one assessable extramedullary disease. Although in previous studies, the incidence of EM-B was higher than that of EM-E, the incidence of EM-E was higher in our study. This might be because some EM-B patients received radiotherapy and were ineligible for this humanized anti-BCMA CAR T cell clinical trial.

There are currently no guidelines regarding treatment for EMM. The outcomes of patients with EMM are different from those of MM without extramedullary disease, even those who receive the same therapy (9). Therefore, the prognosis of primary and secondary EMM is very poor (30). A study on the different prognoses of EMM patients showed that the OS of patients with EM-E (5 months) was poorer than that of patients with EM-B (12 months) (32). In another retrospective series on secondary EMM, the OS were 13.6 months for EM-E and 39.8 months for EM-B (33). In our study, the mean OS of patients with EMM was 17.29 ± 4.95 months. Whether humanized anti-BCMA CAR T-cell therapy has a better efficacy in R/R MM patients with extramedullary disease and prolongs the OS requires further exploration. A recent retrospective study showed that auto-HSCT imparted a survival benefit for patients with both EM-E and EM-B (33). The median PFS from diagnosis was 49 months in EMM patients who received auto-HSCT and 28.1 months in EMM patients who did not receive auto-HSCT. However, few other studies have shown that auto-HSCT could overcome the poor prognosis of EMM. Most studies have consistently shown that the prognosis for EMM is very poor, even among patients who undergo auto-HSCT (30, 34, 35). In our study, two patients (Pt_with_ 1 and 5) who received auto-HSCT did not benefit from this therapy.

BCMA is a highly selective target for CAR T-cell therapy in patients with R/R MM (36). This therapy has emerged as a novel therapeutic approach with the potential for long-term disease control in R/R MM patients (17–20, 37). In particular, humanized anti-BCMA CAR T cell therapy, which has been approved by the FDA (37), had an ORR of 85% in 33 R/R MM patients. This included 15 patients (45%) who achieved CR. However, 6 of the 15 patients who had CR relapsed. Hematologic toxicity and CRS were the most common AEs. However, very few studies have focused on the evaluation of anti-BCMA CAR T-cell therapy in R/R MM patients with EM-E or EM-B. In Brudno’s study of anti-BCMA CAR T cell therapy, one R/R MM patient with an abdominal mass was reported to be responsive to this therapy (38). Another study reported a clinical trial of a biepitope-targeting CAR against BCMA (LCAR-B38M), in which five R/R MM patients with extramedullary infiltration experienced tumor disappearance after CAR T cell therapy. One R/R MM patient with EM-B showed absence of extramedullary disease and malignant pleural effusion after anti-BCMA CAR T cell therapy in another study (18).

Although there was no difference in the PFS and OS rates in R/R MM patients with and without extramedullary disease at 180 days, the PFS and OS rates in patients with extramedullary disease were lower than those in patients without extramedullary disease at 360 days. Therefore, the long-term efficacy of anti-BCMA CAR T cell therapy in R/R MM patients with extramedullary disease remains unsatisfactory. It is whether these patients benefit from radiotherapy or HSCT before further disease progression. We need to explore this issue further. Another interesting finding is that some patients with extramedullary disease experienced further disease progression, but the level of M protein did not increase. However, this phenomenon was not observed in R/R MM patients without extramedullary disease. In the process of observation after their anti-BCMA CAR-T cell therapy, the examination to R/R MM patients with extramedullary disease should be comprehensive, rather than just observing the changes of M protein levels.

CAR T cell therapy can produce potentially life-threatening toxicities, such as CRS and neurotoxicity (23, 39–41). Close monitoring of the side effects of CAR T cell therapy can help control such complications with prompt and appropriate treatment (42–46). Although there was no difference in the median expansion peak of the humanized anti-BCMA CAR T cells in the two patient groups in our study, the grades of CRS and ICANS in patients with extramedullary disease were higher than those in patients without extramedullary disease among patients who responded to therapy. Further, the serum IL-6 was much higher in patients with extramedullary disease. These results might be related to the tumor burden in patients with extramedullary disease. Therefore, attention should be paid to AEs in R/R MM patients with extramedullary disease receiving anti-BCMA CAR T cell therapy.

The efficacy and safety of humanized anti-BCMA CAR T cell therapy in R/R MM patients with extramedullary disease needs to be explored in the future. In particular, anti-BCMA CAR T cell therapy is used in combination with additional therapies to improve efficacy. We might be able to improve the outcomes of R/R MM patients with extramedullary disease who respond to anti-BCMA CAR T cell therapy by bridging to transplantation or radiotherapy before further disease progression.

## Conclusion

The monitoring to R/R MM patients with extramedullary disease after the anti-BCMA CAR-T cell therapy should be comprehensive. Although the grades of CRS and ICANS were much higher in patients with extramedullary disease, the ORR was not different in patients with or without extramedullary disease, and there was no difference in the PFS and OS rates between the two groups at 180 days. These results indicate an opportunity for R/R MM patients with extramedullary disease who respond to anti-BCMA CAR T-cell therapy to bridge to other treatments.

## Data Availability Statement

The original contributions presented in the study are included in the article/**Supplementary Material**. Further inquiries can be directed to the corresponding authors.

## Ethics Statement

The studies involving human participants were reviewed and approved by the Medical Ethics Committee of the Department of Hematology, Tianjin First Center Hospital (Tianjin, China) (Approved No. of ethic committee: 2015002X and 2020N028KY). The patients/participants provided their written informed consent to participate in this study. Written informed consent was obtained from the individual(s) for the publication of any potentially identifiable images or data included in this article.

## Author Contributions

Concept and design: QD and YW. Drafted or revised the manuscript: HD, ML, TY, HZ, XW, and RC. Acquisition of data: HD, JY, and JL. Analysis and interpretation of data: HD and QD. Writing, review, and/or revision of manuscript: HD. Study supervision: QD. All authors contributed to the article and approved the submitted version.

## Conflict of Interest

Author JY was employed by the company Shanghai Genbase Biotechnology Co., Ltd.

The remaining authors declare that the research was conducted in the absence of any commercial or financial relationships that could be construed as a potential conflict of interest

## Publisher’s Note

All claims expressed in this article are solely those of the authors and do not necessarily represent those of their affiliated organizations, or those of the publisher, the editors and the reviewers. Any product that may be evaluated in this article, or claim that may be made by its manufacturer, is not guaranteed or endorsed by the publisher.
