# Supplementary figures and images for: Efficacy of Humanized Anti-BCMA CAR T Cell Therapy in Relapsed/Refractory Multiple Myeloma Patients With and Without Extramedullary Disease
Source: Front Immunol. 2021 Aug 5;12:720571. doi: 10.3389/fimmu.2021.720571 (PMC8374046; doi:10.3389/fimmu.2021.720571)

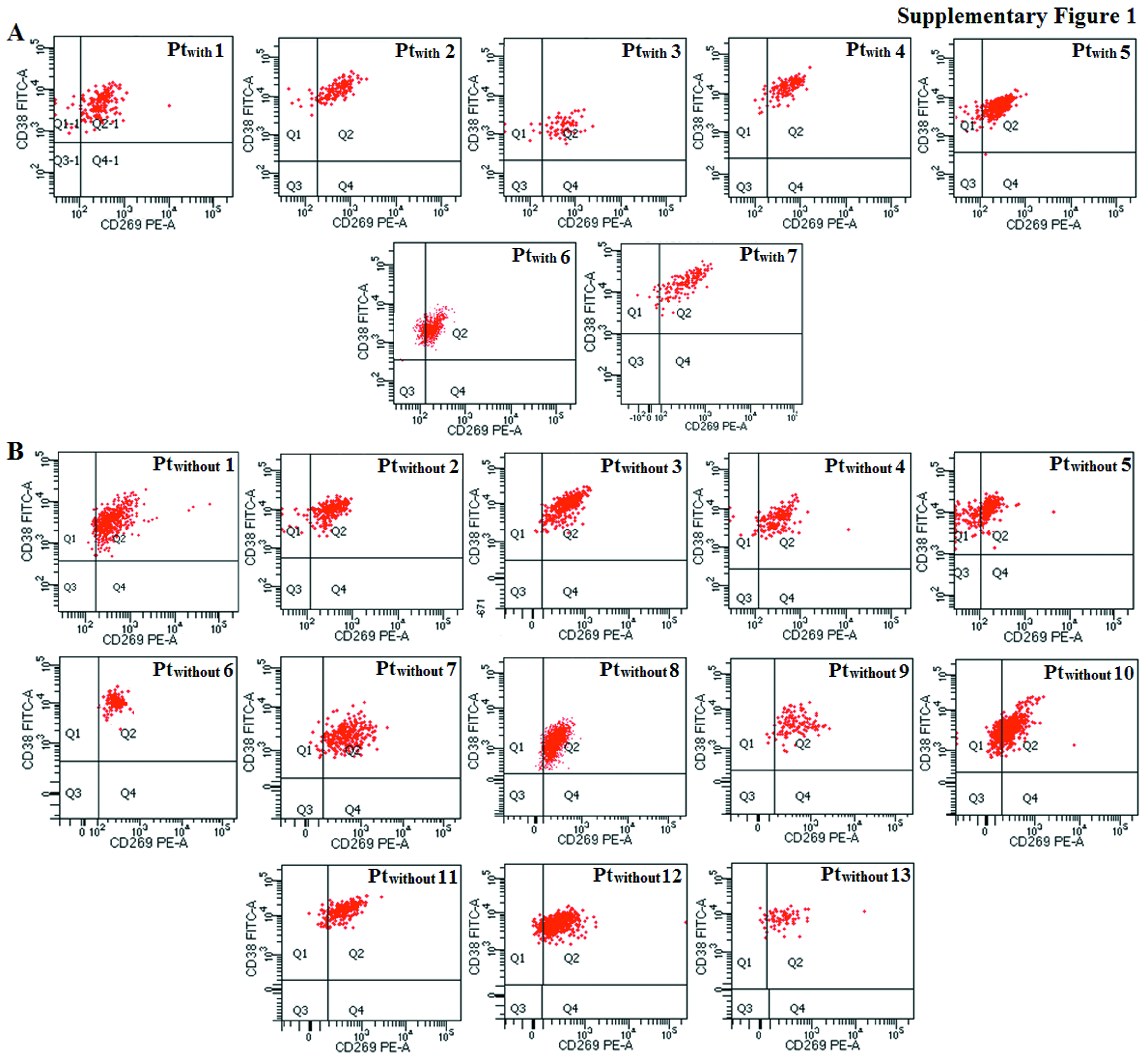

Supplement: Supplementary file 1 [file Image_1.tif]
