# Supplementary material for: Efficacy of Humanized Anti-BCMA CAR T Cell Therapy in Relapsed/Refractory Multiple Myeloma Patients With and Without Extramedullary Disease
Source: Front Immunol. 2021 Aug 5;12:720571. doi: 10.3389/fimmu.2021.720571 (PMC8374046; doi:10.3389/fimmu.2021.720571)
Supplement: Supplementary file 2 [file Table_1.docx]

**Supplementary table 1.**

Manufactured products and phenotypical analysis of the final formulation per patient.

| **Pt** | **Transduction efficiency (%)** | **Amplification efficiency (×10^6^ cells /kg)** | **Infusion dose**  **(×10^6^ cells /kg)** | **Cell harvesting time (Days)** | |
| --- | --- | --- | --- | --- | --- |
| ***With extramedullary disease*** | | | | |  |
| **1** | 36.5 | 7.52 | 2.39 | 12 | |
| **2** | 42.6 | 2.84 | 1.67 | 14 | |
| **3** | 47.6 | 3.52 | 2.34 | 12 | |
| **4** | 23.7 | 8.96 | 2.07 | 12 | |
| **5** | 28.6 | 9.12 | 2.83 | 12 | |
| **6** | 44.3 | 6.74 | 2.42 | 12 | |
| **7** | 48.2 | 2.39 | 1.78 | 15 | |
| Without extramedullary disease | | | | |  |
| **1** | 59.9 | 6.32 | 2.43 | 12 | |
| **2** | 46.7 | 4.69 | 2.06 | 12 | |
| **3** | 35.6 | 5.36 | 2.71 | 12 | |
| **4** | 31.6 | 3.05 | 1.99 | 13 | |
| **5** | 34.2 | 7.81 | 2.04 | 12 | |
| **6** | 29.3 | 7.95 | 2.31 | 12 | |
| **7** | 56.9 | 5.66 | 2.08 | 12 | |
| **8** | 43.43 | 3.18 | 1.78 | 13 | |
| **9** | 38.5 | 7.31 | 2.46 | 12 | |
| **10** | 57.7 | 2.34 | 1.76 | 15 | |
| **11** | 39.1 | 2.24 | 1.75 | 13 | |
| **12** | 30.1 | 4.46 | 1.87 | 12 | |
| **13** | 46.4 | 5.68 | 1.89 | 12 | |
